# Metabolic engineering of *Saccharomyces cerevisiae* for efficient production of glucaric acid at high titer

**DOI:** 10.1186/s12934-018-0914-y

**Published:** 2018-05-05

**Authors:** Na Chen, Jingya Wang, Yunying Zhao, Yu Deng

**Affiliations:** 1 National Engineering Laboratory for Cereal Fermentation Technology (NELCF), Jiangnan University, 1800 Lihu Road, Wuxi, 214122 Jiangsu China; 2 School of Biotechnology, Jiangnan University, 1800 Lihu Road, Wuxi, 214122 Jiangsu China

**Keywords:** Glucaric acid, Metabolic engineering, *Saccharomyces cerevisiae*, *miox4*, Delta-sequence integration

## Abstract

**Background:**

Glucaric acid is a high-value-added chemical that can be used in various fields. Because chemical oxidation of glucose to produce glucaric acid is not environmentally friendly, microbial production has attracted increasing interest recently. Biological pathways to synthesize glucaric acid from glucose in both *Escherichia coli* and *Saccharomyces cerevisiae* by co-expression of genes encoding *myo*-inositol-1-phosphate synthase (Ino1), *myo*-inositol oxygenase (MIOX), and uronate dehydrogenase (Udh) have been constructed. However, low activity and instability of MIOX from *Mus musculus* was proved to be the bottleneck in this pathway.

**Results:**

A more stable *miox4* from *Arabidopsis thaliana* was chosen in the present study. In addition, high copy delta-sequence integration of *miox4* into the *S. cerevisiae* genome was performed to increase its expression level further. Enzymatic assay and quantitative real-time PCR analysis revealed that delta-sequence-based integrative expression increased MIOX4 activity and stability, thus increasing glucaric acid titer about eight times over that of episomal expression. By fed-batch fermentation supplemented with 60 mM (10.8 g/L) inositol, the multi-copy integrative expression *S. cerevisiae* strain produced 6 g/L (28.6 mM) glucaric acid from *myo*-inositol, the highest titer that had been ever reported in *S. cerevisiae*.

**Conclusions:**

In this study, glucaric acid titer was increased to 6 g/L in *S. cerevisiae* by integrating the *miox4* gene from *A. thaliana* and the *udh* gene from *Pseudomonas syringae* into the delta sequence of genomes. Delta-sequence-based integrative expression increased both the number of target gene copies and their stabilities. This approach could be used for a wide range of metabolic pathway engineering applications with *S. cerevisiae*.

**Electronic supplementary material:**

The online version of this article (10.1186/s12934-018-0914-y) contains supplementary material, which is available to authorized users.

## Background

Glucaric acid, which was one of the “top value-added chemicals from biomass” announced by the US Department of Energy in 2004 [1], has been studied for therapeutic uses in cholesterol reduction [2] and cancer therapy [3] as well as for use as a building block for polymers like nylon [4]. Currently, glucaric acid is produced mainly by chemical oxidation of glucose, with nitric acid as solvent and oxidant [5], which has led to low yields and toxic byproducts. Therefore researchers have been showing more interest in microbial production of glucaric acid in an effective and environmentally friendly manner.

Moon et al. [1] constructed a biological pathway to produce glucaric acid from glucose in *Escherichia coli* by co-expression of genes encoding *myo*-inositol-1-phosphate synthase (Ino1) from *Saccharomyces cerevisiae*, *myo*-inositol oxygenase (MIOX) from *Mus musculus*, and uronate dehydrogenase (Udh) from *Pseudomonas syringae*. Analysis of this metabolic pathway revealed that MIOX was rate-limiting, leading to a low glucaric acid titer of 0.72 g/L. To increase d-glucaric acid titer in *E. coli*, novel synthetic tools like synthetic scaffolds and a N-terminal SUMO fusion were used to increase MIOX activity. These two engineered strains produced 2.5 and 4.75 g/L d-glucaric acid from 10 g/L glucose and 60 mM *myo*-inositol [6, 7]. However, it appeared that a d-glucaric acid titer above 5 g/L would inhibit further production in *E. coli* due to a pH-mediated effect. Gupta et al. [8] transferred the synthetic glucaric acid pathway from *E. coli* to *S. cerevisiae* and found that *myo*-inositol availability for the At strain (*S. cerevisiae* containing *miox4* gene from *Arabidopsis thaliana*) and MIOX activity for the Mm strain (*S. cerevisiae* containing MIOX from *M. musculus*) were rate-limiting. Maximum titer in the At strain was 1.6 g/L glucaric acid from glucose supplemented with *myo*-inositol. Liu et al. [9] constructed a glucaric acid synthetic pathway in *Pichia pastoris* using MIOX from *P. pastoris* itself or mice and Udh from *Pseudomonas putida*, resulting in glucaric acid titers of 107.19 ± 11.91 and 785.4 ± 1.41 mg/L respectively.

**Fig. 1:**
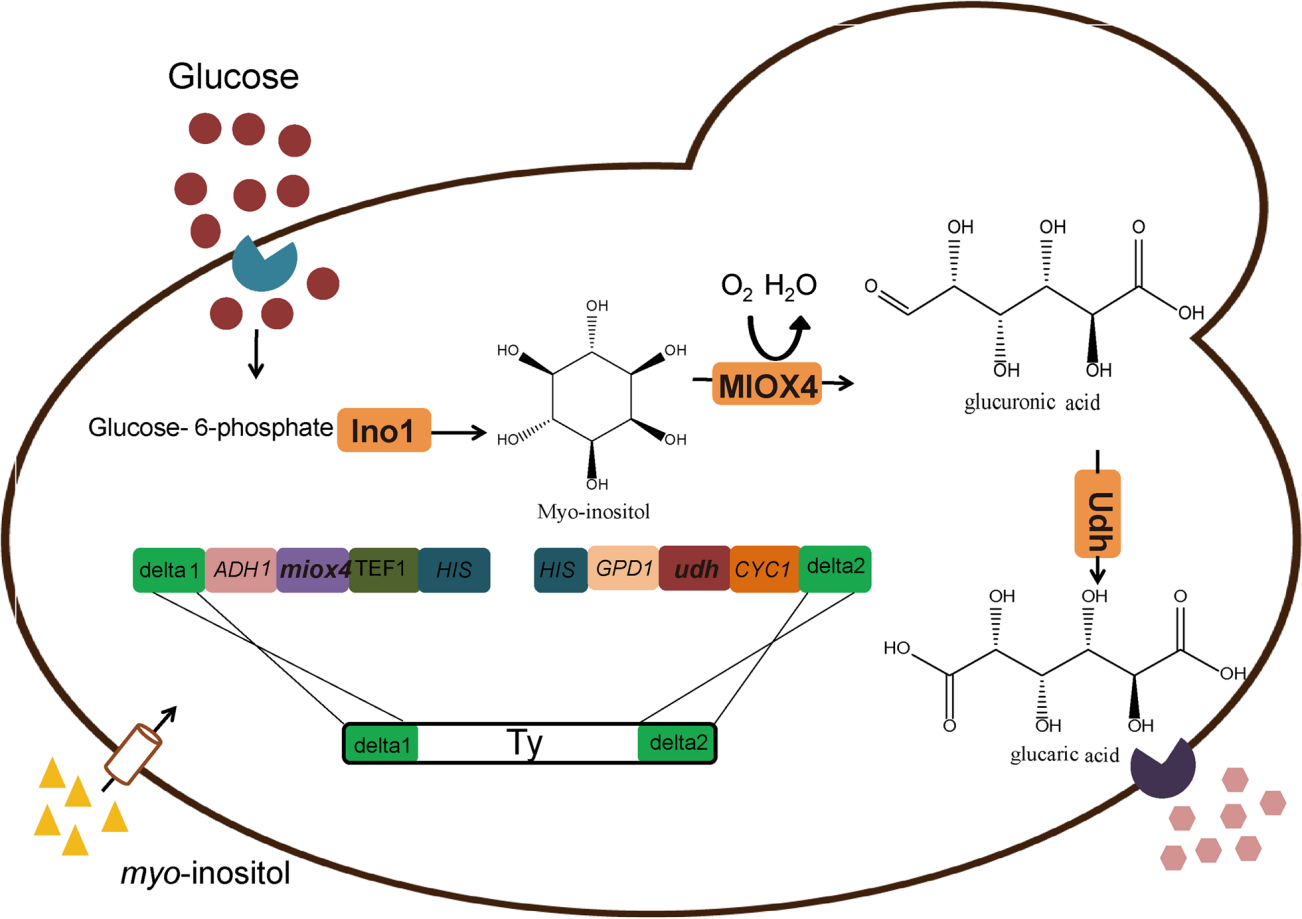
Biosynthetic pathway and integration strategy of *miox4* and *udh* for over-producing glucaric acid in *Saccharomyces cerevisiae*. PCR-amplified products including *miox4* and *udh* genes controlled by strong promoters were integrated into the delta locus with over-lapping *HIS3* homology. *INO1*, *myo*-inositol-1-phosphate synthase gene; *miox4*, *myo*-inositol oxygenase gene; *udh*, uronate dehydrogenase gene; *delta1* and *delta2*, multi-copy DNA sequence located in the reverse transcription transposon Ty of the chromosome DNA in *S. cerevisiae*. *ADH1*, *ADH1* terminator; *TEF1*, *TEF1* promoter; *HIS*, *HIS3* selection marker gene; *GPD1*, *GPD1* promoter; *CYC1*, *CYC1* terminator

Considering the relatively low glucaric acid titers in *E. coli* and *S. cerevisiae*, this study aimed to increase glucaric acid titer in *S. cerevisiae* further by integrating *miox4* from *A. thaliana* [10, 11] into a multi-copy delta sequence of genomes. There are at least three reasons for the choice of baker’s yeast: (1) *S. cerevisiae* has better acid tolerance than *E. coli*; (2) as an eukaryotic organism, *S. cerevisiae* has a post-transcription system that may be advantageous for more stable expression of the *miox4* gene derived from *A. thaliana*; and (3) some yeast strains have been widely used in the study of organic compound production both for their safety and their sophisticated genetic engineering toolkit [12]. This toolkit includes the organic acid, malic acid, and succinic acid produced by engineered *S. cerevisiae* [13, 14] and the fatty acid-based fuels and lipids synthesized by *Y. lipolytica* [15, 16]. Delta-sequence-based constitutive expression increased both the number of target gene copies and their stabilities [17]. This approach could be used for a wide range of metabolic pathway engineering applications of *S. cerevisiae* (Fig. 1).

## Methods

### Strains and culture conditions

All strains and plasmids are shown in Table 1, and the primers used in this study are listed in Additional file 1: Table S1. *E. coli* JM109 cells were cultured in LB medium [1% (w/v) tryptone, 0.5% (w/v) yeast extract, and 1% (w/v) NaCl] at 37 °C. Yeast cells were cultured in YPD medium [2% (w/v) tryptone, 1% (w/v) yeast extract, and 2% (w/v) sodium chloride] or SD-URA/SD-HIS medium [0.17% (w/v) yeast nitrogen base, 2% (w/v) glucose, 0.5% ammonia sulfate, adding 1/10 mL amino acid mixture without uracil or histidine] at 30 °C. Solid media were produced by adding 2% (w/v) agar if necessary.

**Table 1:** Strains and plasmids used in this study

Strains and plasmids	Relevant genotype	Reference
*E. coli* JM109		Stored in author’s laboratory
*S. cerevisiae* BY4741	*MATa, his3Δ1, leu2Δ0, met15Δ0, ura3Δ0*	Stored in author’s laboratory
BY4741 *opi1∆* Bga-0	*MATa, his3Δ1, leu2Δ0, met15Δ0, ura3Δ0, opi1∆0* BY474 carrying pY26GPD-TEF-miox4-udh	Stored in author’s laboratory This study
Bga-1	BY474 *opi1∆ c*arrying pY26GPD-TEF-miox4-udh	This study
Bga-2	BY474 *opi1∆* carrying pY26GPD-TEF-miox4-histag	This study
Bga-3	BY474 *opi1∆* carrying *miox4* and *udh*	This study
*E. coli* pUG6	*E. coli* plasmid with segment LoxP-KanMX4-LoxP	Stored in author’s laboratory
*E. coli* pSH65 pY26-GPD-TEF pY26-miox4-udh pY26-miox4-histag	Shuttle plasmid for *E. coli* and *S. cerevisiae*, Cre gene under GAL2 regulative regulation Integrating plasmid, URA3 marker, MCS derived from pBLUESCRIPT (ColE1 (derivative) ori, f1 ori, Amp^R^) pY26GPD-TEF carrying *miox4* and *udh* pY26GPD-TEF carrying *miox4*-histag	Stored in author’s laboratory Stored in author’s laboratory This study This study

For shake flask cultivation, 50 mL of YPD medium with or without 10 g/L *myo*-inositol in a 250 mL shake flask was used as the initial fermentation medium. Cultures were first grown overnight at 30 °C and 250 rpm in YPD medium with *myo*-inositol and then inoculated to a density at 600 nm (OD_600_) of 0.05. Cultures were incubated at 30 °C and 250 rpm for about 7 days.

### Plasmid construction

The *miox4* [10] gene encoding MIOX4 enzyme in *A. thaliana* and the *udh* gene encoding Udh in *P. syringae* [18] were codon-optimized and synthesized by Genewiz (Suzhou, China). To construct the pY26-miox4 plasmid, the *miox4* gene was first digested with *Bgl*II/*Not*I and then ligated to the pY26-GPD-TEF plasmid [19], which was digested with *Bgl*II/*Not*I to generate the pY26-miox4 plasmid. The *udh* gene was ligated to pY26-miox4 using the Gibson Assembly method to construct the pY26-miox4-udh plasmid. The *miox4* gene with 6× his-tag was PCR-amplified, digested with *Eco*RI/*Xho*I, and ligated to *Eco*RI/*Xho*I-digested pY26-GPD-TEF to generate the pY26-miox4-6×His plasmid expressing the MIOX4-6×His fusion protein.

### Genetic manipulations

To knock out the *OPI1* [20, 21] gene, which was functional in the negative regulation of Inositol-1-phosphate synthase (Ino1), the loxP-kan-loxP cassette [22] containing the upstream and downstream 50 bp of the *OPI1* gene was amplified by PUG 6F and PUG 6R primers from the pUG6 plasmid and transformed into *S. cerevisiae* BY4741-competent cells through the Li–Ac method [23]. The kanMX selection cassette was cured from successful deletion mutants using Cre recombinase expressed by the pSH47 [24] plasmid. Then the deletion mutant was subcultured continuously for loss of pSH47, generating the BY4741 *opi1∆* strain. To integrate exogenous genes into the genome, delta1 and delta2 [25, 26] fragments were amplified from the *S. cerevisiae* genome using genF-2/genR-2 and genF-5/genR-5 primers respectively, and the *miox4* and *udh* expression cassettes TMA and GUC were amplified from the pY26-miox4-udh plasmid by genF-3/genR-3 and genF-4/genR-4 primers respectively. In addition, two parts of the selection marker HIS3, HIS3-1 and HIS3-2, were amplified from pRS313 using genF-1/genR-1 and genF-6/genR-6. The above PCR products were gel-extracted, and touchdown PCR was used to assemble two homologous arms containing delta1, TMA, HIS3-1 and HIS3-2, GUC, and delta2. Then the two homologous arms were transformed into a solid medium lacking histidine, and the high glucaric acid-producing strain was screened.

### MIOX4 activity assay and immunoblot analysis

Bga-2 containing pY26-miox4-6×His plasmid was cultured in SD-Ura medium at 30 °C in a rotary shaker at 250 rpm up to OD_600_ of 0.6–0.8, then inoculated into 50 mL of SD-Ura medium using *S. cerevisiae* BY4741 containing pY26-GPD-TEF plasmid as a control. After 24 h cultivation, cell pellets were harvested and re-suspended in 50 mM Tris–HCl buffer (pH 7.0), then disrupted by ultrasonication and centrifuged at 13,000×*g* for 10 min. The supernatant was used for the enzymatic activity assay. The reaction system (200 μL) contained 50 mM Tris–HCl buffer (pH 7.0), 2.0 mM l-cysteine, 1.0 mM ammonium ferrous sulfate, 60 mM (10.8 g/L) *myo*-inositol, and a certain amount of enzyme. The reaction system without *myo*-inositol was pre-heated at 30 °C for 10 min and then proceeded at 30 °C, 200 rpm for 1 h after addition of *myo*-inositol. Then the reaction was stopped by boiling for 15 min, and the supernatant was extracted after centrifugation at 12,000×*g* for 10 min. The glucuronic acid concentration in the supernatant was determined by a chromogenic method using orcinol reagent (100 mL of 37% HCl, 0.1 g of orcinol, and 0.1 g of ferric trichloride). The supernatant and two times the volume of orcinol reagent were boiled together for 30 min and cooled to room temperature, after which OD_660_ was measured to quantify the glucuronic acid concentration [27]. The reaction system without *myo*-inositol was set as a control to determine MIOX4 activity. The protein concentration was determined by the Bradford method using BSA as a standard. One unit of MIOX4 was defined as the enzyme content needed to convert 1 μmol *myo*-inositol in 1 min [28–30].

To analyze the expression level of MIOX4 protein, the Bga-2 recombinant strain was cultured in YPD medium to OD_600_ = 0.8, then inoculated into 50 mL of YPD medium and cultured for 48 h. The whole cell protein was extracted to perform a Western blot, after which 15 μg of total protein from each lysate was separated by 10% SDS-PAGE and transformed onto PVDF membranes (Sangon Biotech, Shanghai, China). After blotting, the membranes were incubated overnight in a 1:2000 dilution of anti-HIS antibody according to the manufacturer’s instructions. Western blot analysis was carried out as described previously [31].

### Relative quantification of gene expression levels

To quantify gene expression levels, samples of approximately 10^8^ cells were harvested after inoculation for 16 h. Total RNA was extracted from each of the samples using the RNAiso Plus RNA isolation kit (TaKaRa, Dalian, China) after liquid nitrogen grinding according to the manufacturer’s instructions. Following genomic DNA eraser treatment to remove DNA, cDNA was synthesized from 500 ng of total RNA using random primers with the PrimerScript RT reagent kit. The synthesized cDNA was then amplified in a quantitative PCR (qPCR) with primers Miox-a/Miox-s, Udh-a/Udh-s, and Pgk-a/Pgk-s respectively for relative quantification of mRNA levels of *miox4* and *udh*, using the phosphoglucokinase gene (*PGK1*) as an internal control. qPCR reactions containing SYBR Premix Ex Taq were performed in a Thermo Scientific CFX96 instrument. Each reaction was carried out in triplicate, and the reported Ct value was the average of the triplicate samples. Transcript levels were calculated using the − ΔΔCt method [32].

### Fed-batch fermentation and metabolite analysis

Slant culture was inoculated into 60 mL YPD medium and cultured at 30 °C, 250 rpm for 24 h to the exponential growth phase. Before fermentation, the rotation speed, ventilation volume, and DO were set to 600 rpm, 3 L/min, and 100% respectively. Then 60 mL seed culture was inoculated into a 5-L bioreactor containing 3 L fermentation medium, where pH was controlled at 4 ± 0.1 using 2.0 M HCl and 2.0 M NaOH and DO was controlled at 50% at 30 °C.

Fermentation samples were taken, filtered through a 0.22-μm membrane, and subjected to high-performance liquid chromatography (HPLC). Glucaric acid, lactic acid, ethanol, acetic acid, and glucose concentration were quantified by a Hitachi Chromaster using an Aminex HPX-87H column (300 mm × 7.8 mm; Bio-Rad Laboratories, Hercules, CA). Sulfuric acid (5 mM) was used as the mobile phase at 30 °C and a flow rate of 0.6 mL/min in isocratic mode. Compounds were detected from 30-µL injections using a refractive index detector.

## Results

### Construction of the biosynthetic pathway to produce glucaric acid in *Saccharomyces cerevisiae*

**Fig. 2:**
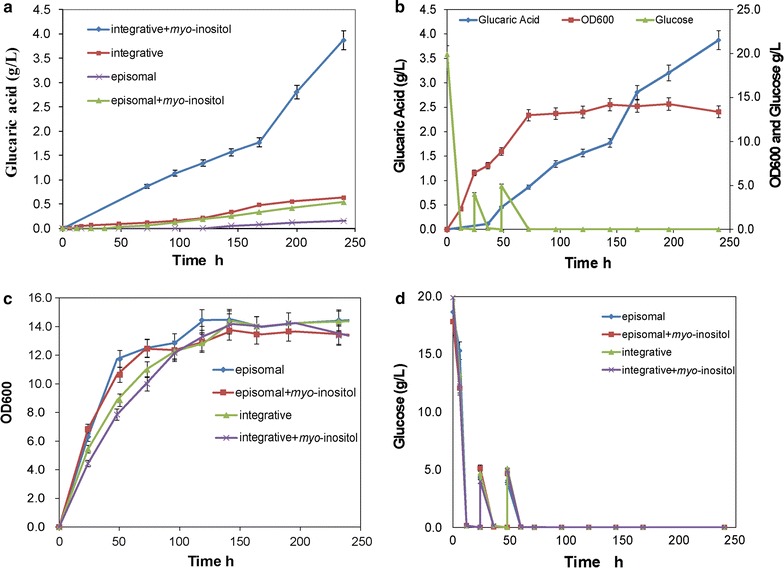
Fermentation results for glucaric acid by episomal and integrative expression strains. **a** Glucaric acid titer of episomal and integrative expression strains in YPD medium with and without *myo*-inositol. **b** Glucaric acid production of the integrative strain (Bga-3) in YPD medium with *myo*-inositol. **c** Growth of episomal and integrative expression strains in YPD medium with and without *myo*-inositol. **d** Glucose consumption of episomal and integrative expression strains in YPD medium with and without *myo*-inositol. All experiments were performed in triplicate, and the error bars represent mean ± standard deviation

To construct the glucaric acid biosynthetic pathway, a pY26-miox4-udh plasmid expressing *miox4* and *udh* genes under control of strong promoters GPD and TEF [33] respectively was constructed and transformed into *S. cerevisiae* strain BY4741 *opi1∆*, generating strain Bga-1. The Bga-1 strain was cultivated in YPD medium supplemented with 60 mM (10.8 g/L) *myo*-inositol [34], where glucaric acid should be detected if *miox4* and *udh* were both successfully expressed. As expected, glucaric acid was successfully detected in the culture supernatant of the Bga-1 strain by LC–MS. The highest production of glucaric acid was 0.54 g/L in YPD medium containing 60 mM (10.8 g/L) *myo*-inositol after 240-h shake flask cultivation (Fig. 2a). However, no glucaric acid was produced when no *myo*-inositol was supplemented into the culture medium, which indicated that the *myo*-inositol produced by the yeast cell itself was not enough to produce glucaric acid.

### *miox4* derived from *Arabidopsis thaliana*

**Fig. 3:**
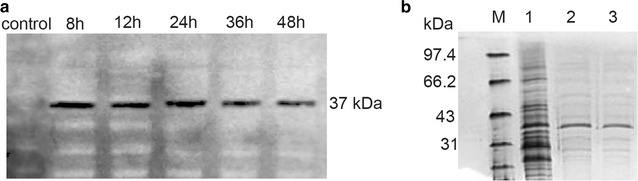
MIOX4 protein analysis. **a** Western blot analysis of MIOX4. Whole-cell protein was extracted to perform Western blot after the recombinant strain Bga-2 was cultured in YPD medium with 60 mM (10.8 g/L) *myo*-inositol for the indicated time. **b** SDS-PAGE analysis of MIOX4. To purify the MIOX4 protein, the His-tag was fused to the C-terminal of MIOX4. Strains were cultured as above, and the whole-cell protein was extracted and purified with the 6× His-Tagged Protein Purification Kit. The purified protein was verified by SDS-PAGE. Lane 1: protein marker, Lane 2: control (BY4741 carrying pY26 plasmid), Lanes 3 and 4: Bga-2

MIOX was first reported in 1957, when Charalampous et al. [27] found that one kind of enzyme that can convert *myo*-inositol to glucuronic acid existed in the mouse kidney. Reddy et al. [28, 29] isolated MIOX from hog kidney and found that MIOX could be activated after incubation in Fe^2+^ and cysteine, which solved the instability problem for this enzyme [9]. In addition, Moon et al. [1] synthesized the gene sequence of mouse-derived MIOX, cloned it into pTrc plasmid, and transformed it into *E. coli* BL21 (DE3) for expression. Although enzyme activity reached 0.43 U/mg, MIOX was found to be extremely unstable. Activity reached a maximum after culturing in a medium containing 60 mM (10.8 g/L) of *myo*-inositol, but then sharply declined, making MIOX a rate-limiting step in the glucaric acid synthetic pathway. Gupta et al. constructed a glucaric acid synthetic pathway in *S. cerevisiae* using MIOX from both *M. musculus* (Mm strain) and *A. thaliana* (At strain). They found that the At strain produced more glucaric acid (0.56 g/L) than the Mm strain (0.29 g/L), but glucaric acid production was still limited by both MIOX activity and *myo*-inositol availability during various growth stages. The authors of this paper believe that MIOX from *A. thaliana* is more stable than that from *M. musculus* [8]. Therefore, the more stable *myo*-inositol oxygenase MIOX4 [10] derived from *A. thaliana* was chosen in this study. To confirm MIOX4 activity, the pY26-miox4-histag plasmid was constructed, which could express the MIOX4-6×His fusion protein and transform it to BY4741 *opi1∆*, generating the Bga-2 strain. Western blot analysis revealed that the MIOX4 protein was successfully expressed in *S. cerevisiae* with a molecular weight of 37 kDa [34] (Fig. 3a). MIOX4 protein purification [35] by a Ni-column affinity chromatography kit also indicated that MIOX4 was successfully expressed in *S. cerevisiae* (Fig. 3b). After deletion of the *OPI1* gene, *S. cerevisiae* accumulated 0.4 g/L of *myo*-inositol, and the MIOX activity was much higher in the *opi1* mutant than that of the wild-type cells (Additional file 1: Figure S1), which indicated that the deletion of *OPI1* gene played a significant role in maintaining MIOX4 activity [20, 21].

**Fig. 4:**
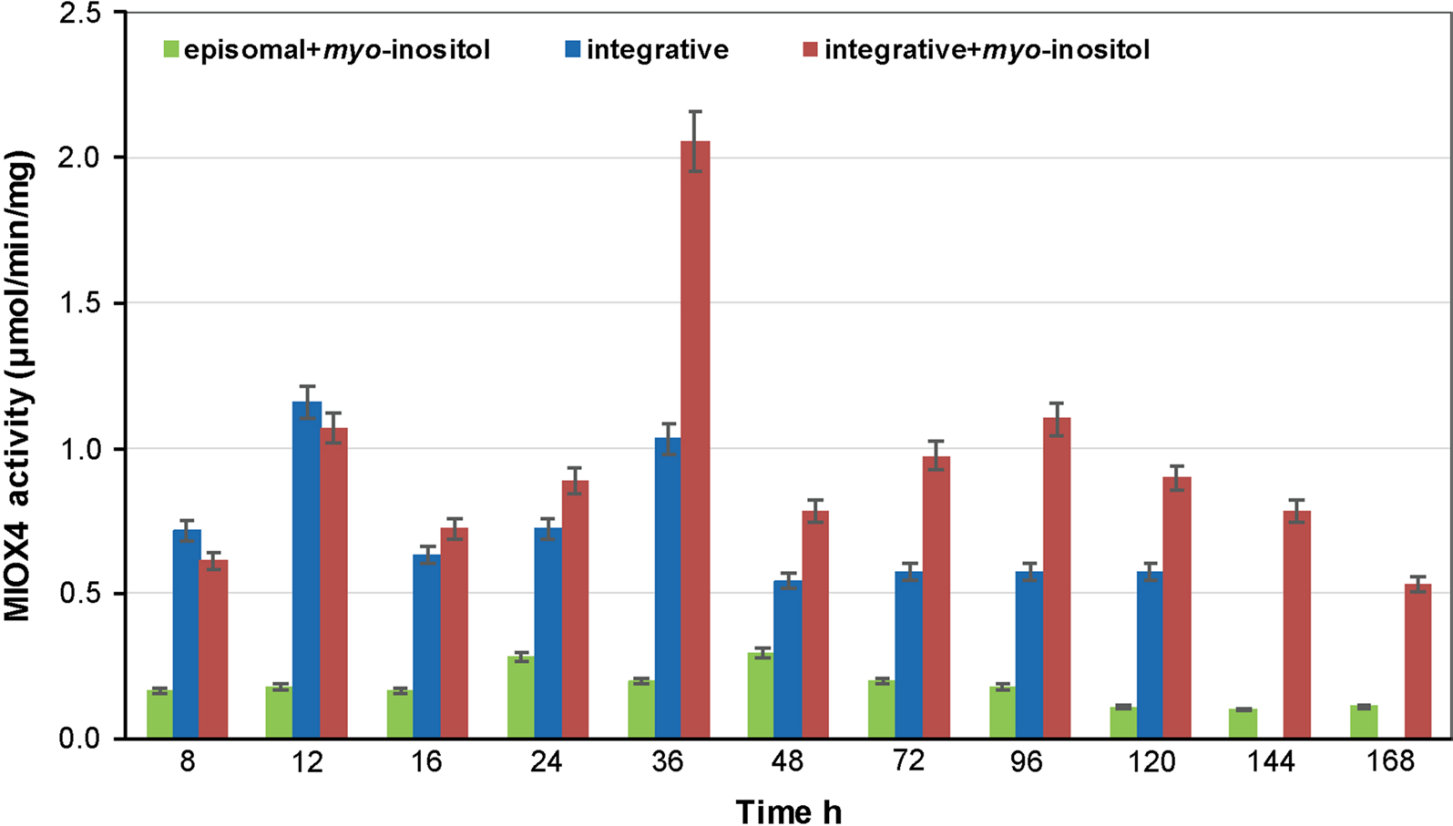
MIOX4 activity in the episomal expression strain with *myo*-inositol and the integrative expression with and without *myo*-inositol. All experiments were performed in triplicate, and the error bars represent mean ± standard deviation

The BY4741 *S. cerevisiae* strain containing pY26-miox4-histag was cultured in YPD medium for 7 days. The protein was extracted, adjusted to the desired concentration, and subjected to a Western blot test. MIOX4 protein expression decreased slightly in 36 h (Fig. 3a), whereas the enzymatic activity of MIOX4 declined after 48 h of MIOX4 protein expression, but became stable in the following 6 days (Fig. 4). These results showed that *A. thaliana*-derived MIOX4 was more efficient and stable in *S. cerevisiae* than *M. musculus*-derived MIOX expressed in *E. coli* [1].

### Increased glucaric acid titer through delta-sequence-based integrative expression

**Fig. 5:**
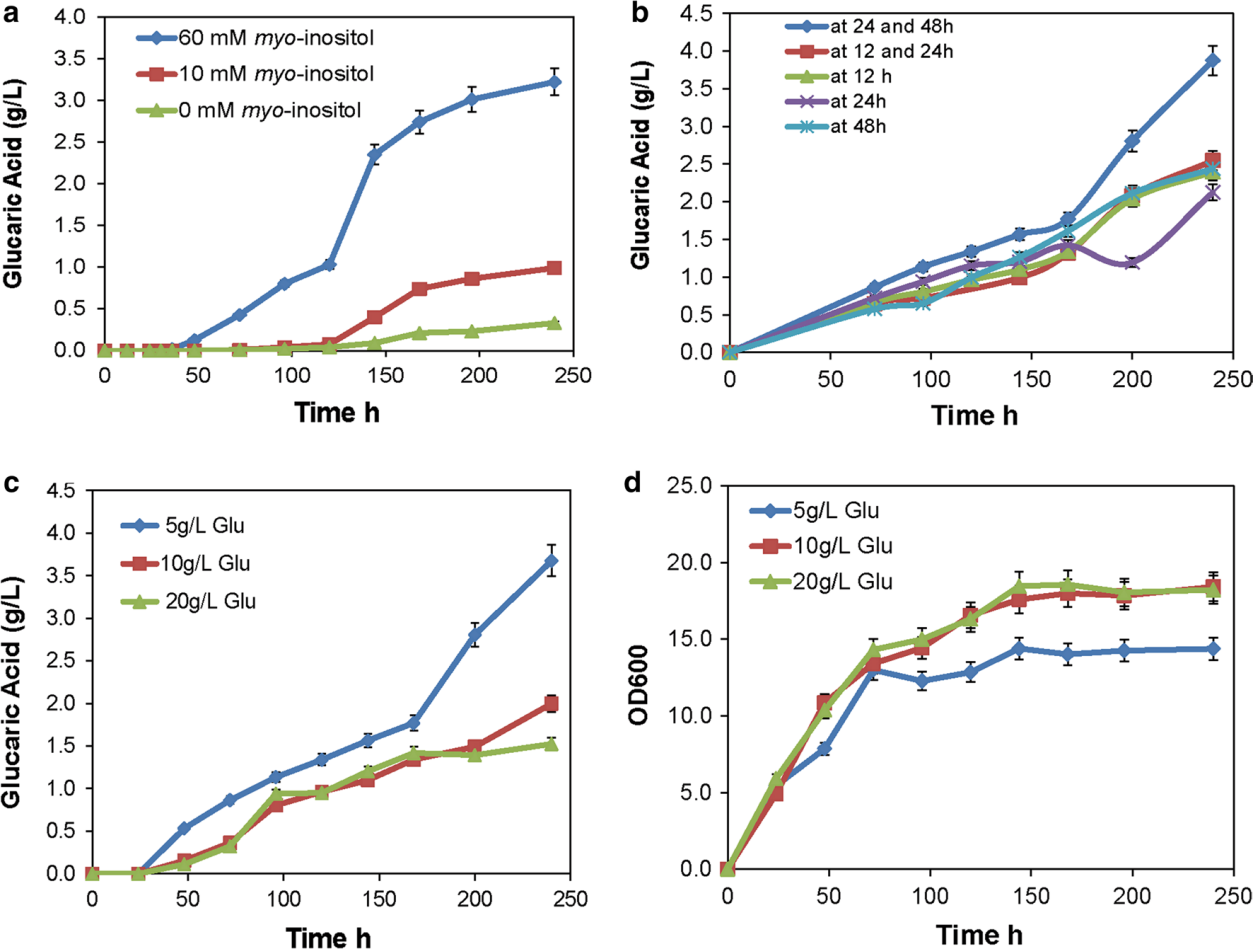
Fermentation results for glucaric acid by strain Bga-3. **a** Glucaric acid produced in YPD medium with variable *myo*-inositol concentrations. **b** Glucaric acid produced in YPD medium supplemented with 60 mM (10.8 g/L) *myo*-inositol at different points in time. **c** Glucaric acid produced in YPD medium with variable glucose concentrations. **d** Growth profile of Bga-3 in YPD medium with variable glucose concentrations. All experiments were performed in triplicate, and the error bars represent mean ± standard deviation

It was suspected that the main reason for low glucaric acid titer was the lower expression level of *miox4* and *udh* genes in plasmid episomal expression, especially the *miox4* gene. To circumvent plasmid instability and increase the number of target gene copies, a delta sequence-based constitutive expression method was used to overexpress target genes [25]. *S. cerevisiae* delta sequences are long terminal repeats of transposons Ty1 and Ty2, and a total of 425 delta sequences are dispersed through the yeast genome [17]. *miox4* and *udh* were integrated under control of GPD and TEF promoters [33] respectively into the chromosome of *S. cerevisiae* BY4741 *opi1∆* through delta sequence-based homologous recombination (Fig. 1). Following successful integration, one strain with the highest titer was selected and named *S. cerevisiae* BY4741 *opi1∆*-*mu* (Bga-3). This strain produced 3.8 g/L (18.1 mM) glucaric acid in shake flask culture with 60 mM (10.8 g/L) *myo*-inositol, which was 7.04 times higher than that of the Bga-2 episomal plasmid expression in *S. cerevisiae* (Fig. 2b). Interestingly, there were no obvious differences in growth rate or glucose consumption between the Bga-2 episomal plasmid expression strain and the Bga-3 integrative expression strain with or without *myo*-inositol (Fig. 2c, d). YPD medium supplemented with 0, 10 mM (1.8 g/L) or 60 mM (10.8 g/L) of *myo*-inositol was used to produce glucaric acid. The results showed the highest glucaric acid titer generated in 60 mM (10.8 g/L) *myo*-inositol (Fig. 5a), suggesting that *myo*-inositol availability was rate-limiting in glucaric acid synthesis. Then 5 g/L of glucose was fed once at 12, 24, 48 h respectively, or fed twice at 12/24 and 24/48 h. The results showed that glucose fed twice at 24/48 h was best for increasing glucaric acid production (Fig. 5b). Henry et al. [36] reported that the exponential growth phase was the best time for *myo*-inositol accumulation, which further increased glucaric acid titer. The glucose feeding concentration was also tested. 5, 10, and 20 g/L of glucose was fed at 24 and 48 h; the results showed that the highest glucaric acid titer occurred at 5 g/L of glucose, but that higher glucose feeding contents had a negative effect on glucaric acid titer (Fig. 5c). The reason why more glucose decreased glucaric acid titer was suspected to be that excessive glucose fluxed into the glycolytic pathway to increase biomass, instead of *myo*-inositol (Fig. 5d). Therefore, the best fermentation condition was YPD medium supplemented with 60 mM (10.8 g/L) *myo*-inositol, and with 5 g/L of glucose fed at 24 and 48 h. Under this culture condition, the highest glucaric acid titer [3.8 g/L (18.1 mM)] was produced in the shake flask, with a 1.76 ± 0.08 g/g-*myo*-inositol yield and a 0.127 ± 0.005 g/g-glucose yield.

### Increased MIOX4 activity and gene expression level by multi-copy integrative expression

**Fig. 6:**
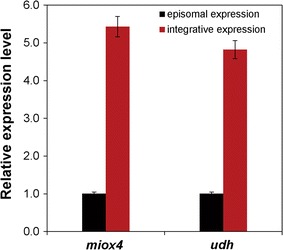
Relative gene expression levels of *miox4* and *udh*. All experiments were performed in triplicate, and the error bars represent mean ± standard deviation

To determine whether the high glucaric acid yield was due to the high expression levels of *miox4* and *udh*, quantitative real-time PCR (RT-qPCR) was performed to confirm whether delta sequence-based integrative expression increased gene expression levels of *miox4* and *udh* compared with episomal plasmid expression. The result showed that the transcription levels of *miox4* and *udh* from integrative expression were 5.43 and 4.82 times of those from episomal expression (Fig. 6). MIOX4 enzymatic activities of integrative and episomal expression with and without *myo*-inositol were also measured. The specific activity of MIOX4 in integrative expression with *myo*-inositol reached a maximum of 2.05 U/mg, then declined, but remained above 0.5 U/mg. In contrast, the highest specific activity of MIOX4 in episomal expression with *myo*-inositol was 0.295 U/mg (Fig. 4), suggesting that multi-copy integrative MIOX4 increased enzymatic activity more than episomal expression by increasing mRNA transcription levels. In addition, MIOX4 activity in integrative expression with *myo*-inositol was more efficient and stable than without *myo*-inositol, which confirmed the extreme importance of *myo*-inositol to the activity of *myo*-inositol oxygenase. This study showed that regardless of episomal plasmid expression or integrative expression, the MIOX4 activity decreased steadily, but remained above a certain value. Unlike *E. coli*, *S. cerevisiae* has an endogenous *myo*-inositol accumulation pathway, thus increasing the glucaric acid production.

### Fed-batch fermentation

**Fig. 7:**
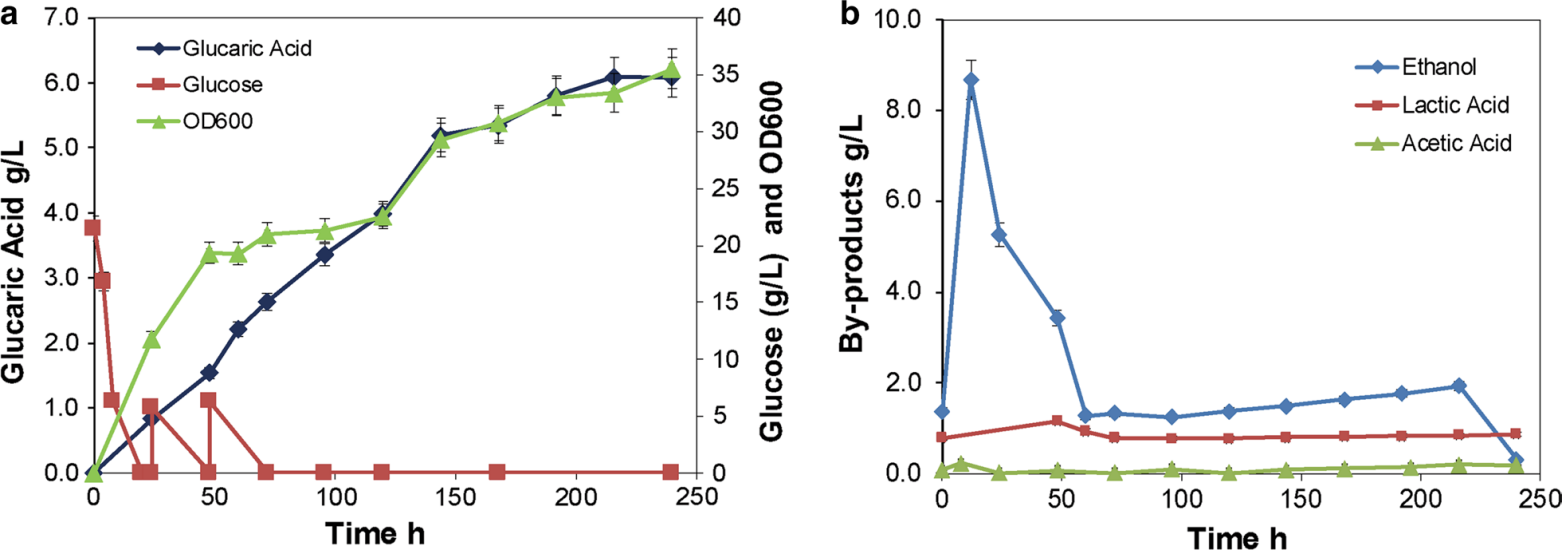
Fed-batch fermentation of glucaric acid by the Bga-3 strain. **a** Growth, glucose consumption, and glucaric acid production. **b** Byproducts production. All experiments were performed in triplicate, and the error bars represent mean ± standard deviation

To provide further confirmation of the performance of the multi-copy episomal expression strain as constructed, fed-batch fermentation in a 5-L bioreactor was carried out. As DO and pH were controlled at their optimal values for *S. cerevisiae* growth, the BY4741 *opi1∆*-mu (Bga-3) strain produced glucaric acid up to 6.0 g/L (28.6 mM) at 216 h (Fig. 7a) with only a few by-products of ethanol, acetic acid, and lactic acid (Fig. 7b). This was the highest titer ever reported in *S. cerevisiae*, with a 1.67 ± 0.08 g/g-*myo*-inositol yield and a 0.2 ± 0.008 g/g-glucose yield. The results obtained showed that the byproduct ethanol and lactic acid accumulated in the initial stage, but declined in the later phase of fermentation. This indicated that the byproduct ethanol and lactic acid may be partially utilized by the yeast cells, as reported previously [37–39].

## Discussion

Cell factories created by metabolic engineering and synthetic biology to produce fine chemicals have emerged as an increasingly popular alternative to chemical synthesis [40]. Ever since the glucaric acid biosynthetic pathway was constructed in *E. coli* [1], great efforts have been expended to increase target product titer. Gupta et al. [8] reported that MIOX activity and *myo*-inositol availability were rate-limiting in glucaric acid production, not only in *E. coli*, but in *S. cerevisiae*. Therefore, the strategy to increase glucaric acid production was to improve MIOX activity and stability while increasing the *myo*-inositol flux to glucaric acid.

In *S. cerevisiae*, *myo*-inositol can be produced by converting d-glucose-6-phosphate by native inositol-1-phosphate synthase (Ino1), which is tightly regulated by Opi1 [36]. In this study, the *OPI1* gene was deleted to remove its negative regulation of *myo*-inositol synthesis. Although more *myo*-inositol was produced by *S. cerevisiae*, exogenous supplementation of *myo*-inositol was still necessary to increase MIOX4 activity (Fig. 2a) and glucaric acid accumulation. In addition, the constructed strain supplemented with 60 mM (10.8 g/L) *myo*-inositol apparently produced more glucaric acid than 10 mM (1.8 g/L) *myo*-inositol (Fig. 5a), which indicated that efficient production of glucaric acid at high titer needs a high concentration of myo-inositol might because the low affinity activity of the Itr1/2 myo-inositol transporter in *S. cerevisiae* [41]. However, only a small proportion of the supplemented *myo*-inositol was transported into the cell (Additional file 1: Figure S2). When the *myo*-inositol residue was fed 60 mM (10.8 g/L) *myo*-inositol to a shake flask culture, only about 20% (2.16 ± 0.11 g/L) of *myo*-inositol was consumed (Additional file 1: Figure S2A). At least 3.26 g/L *myo*-inositol was required to produce 3.8 g/L glucaric acid in the shake flask culture. This means that at least 1.1 g/L *myo*-inositol was endogenously synthesized from glucose; the yields were 1.76 ± 0.08 g glucaric acid/g *myo*-inositol and 0.037 ± 0.003 g *myo*-inositol/g glucose. In the fed-batch fermentation of glucaric acid, about 3.6 ± 0.18 g *myo*-inositol was consumed (Additional file 1: Figure S2B), and about 5.14 g/L *myo*-inositol was required to produce 6.0 g/L glucaric acid. Therefore, at least 1.54 g/L *myo*-inositol came from glucose, and the yield was 1.67 ± 0.08 g glucaric acid/g *myo*-inositol, or 0.051 ± 0.006 g *myo*-inositol/g glucose. Robinson reported that the uptake activity of the Itr1p *myo*-inositol transporter was affected by growth phase in *S. cerevisiae* because it would reach a maximum at exponential and then decrease rapidly to minimum in the stationary phase, and this regulation was independent of *OPI1* [41]. Improving the transport ability of Itr1p through protein engineering may be very important.

Besides *myo*-inositol availability, low activity or instability of *myo*-inositol oxygenase is the key bottleneck in the biosynthetic pathway to glucaric acid. Given that the codon-optimized mouse-derived MIOX gene had low activity and instability according to Moon et al. [1], the *miox4* gene from *A. thaliana* was selected for this study. MIOX4 showed relatively high stability compared with mouse MIOX, but the specific activity of episomal plasmid expression of *miox4* was still very slow. To overcome this problem, delta-sequence-based integrative expression of *miox4* and *udh* genes was carried out. A delta sequence is a kind of long-end repeated DNA sequence located in the reverse transcription transposon Ty of chromosome DNA in *S. cerevisiae*. Because there are approximately 425 delta sequences, delta sequence-based homologous recombination is more efficient than traditional methods for exogenous DNA integration into the *S. cerevisiae* genome, and the target gene was expressed more stably in the delta sequence because it avoided plasmid loss in the episomal expression strain [17]. The *miox4* transcription level as determined by quantitative real-time PCR was an indirect characterization of gene copies compared with that of the episomal plasmid. The transcription level of *miox4* in integrative expression was 5.43 times that of episomal expression (Fig. 6). These results indicated that this multi-copy integrative expression method is efficient for overexpression of *miox4* genes, resulting in obviously enhanced MIOX4 activity and stability and high glucaric acid titer. The authors believe that the delta-sequence integration method can be used in a wide range of low-copy genes to remove the bottleneck in metabolic engineering of *S. cerevisiae*.

## Conclusions

In this study, glucaric acid titer in *S. cerevisiae* was increased by expressing a more stable MIOX4 from *A. thaliana* and integrating the target genes into the delta sequence of the genomes. Delta-sequence-based constitutive expression increased both the number of target gene copies and their stability and can be used for a wide range of metabolic pathway engineering projects in *S. cerevisiae*. The final strain produced 6.0 g/L (28.6 mM) glucaric acid, which is the highest titer reported in *S. cerevisiae*.

## Additional file

**Additional file 1: Table S1.** Primers used in this study. **Figure S1.** MIOX4 activity of the episomal expression plasmid in the wild-type strain and *opi1* mutant strain with *myo*-inositol. All experiments were performed in triplicates and the error bar represented mean ± standard deviation. **Figure S2.** *myo*-inositol residue in shake flask (A) and fed-batch (B) cultures when fed 60 mM (10.8 g/L) *myo*-inositol to the culture. All experiments were performed in triplicates and the error bar represented mean ± standard deviation.
