# Supplementary material for: Metabolic engineering of Saccharomyces cerevisiae for efficient production of glucaric acid at high titer
Source: Microb Cell Fact. 2018 May 5;17:67. doi: 10.1186/s12934-018-0914-y (PMC5935971; doi:10.1186/s12934-018-0914-y)
Supplement: Supplementary file 1 — Additional file 1: Table S1. Primers used in this study. Figure S1. MIOX4 activity of the episomal expression plasmid in the wild-type strain and opi1 mutant strain with myo-inositol. All experiments were performed in triplicates and the error bar represented mean ± standard deviation. Figure S2. myo-inositol residue in shake flask (A) and fed-batch (B) cultures when fed 60 mM (10.8 g/L) myo-inositol to the culture. All experiments were performed in triplicates and the error bar represented mean ± standard deviation. [file 12934_2018_914_MOESM1_ESM.docx]

**Table S1 Primers used in this study**

| primer name | sequense 5’-3’ |
| --- | --- |
| p26mF | AGATCTTCACCAACGCAGATTTTCAG |
| p26mR | GCGGCCGCATGACTATCAGTGTAGAAAA |
| p26muF | GACGGATTCTAGAACTAGTGGATCCCCCGGGCTGCAGGATGGCCTCCG CTCATACTAC GCAAACT |
| p26muR | AGGTCGACGGTATCGATAAGCTTGATATCGAATTTTATTTATCACCAAA  CGGACCACTCGCCACGAA |
| p26mtagF | CG GAATTC ATGACTATCAGTGTAGAAAAG |
| p26mtagR | GGCCTCGAGTCAATGGTGATGGTGATGATGCCAACGCAGATTTTCAGGGAA |
| veri-pYF  veri-pYR  PrKO-*OPI1*F  PrKO-*OPI1*R  *OPI1*kochkF  *OPI1*kochkR  genF-1 | CTGGCAAGGTAGACAAGCCGACAA  GGAGGGCGTGAATGTAAGCGTGA  TTAAAGCGTGTGTATCAGGACAGTGTTTTTAACGAAGATACTAGTCATTGCAGCTGAAGCTTCGTACGC  TATAATATTATTACTGGTGGTAATGCATGAAAGACCTCAATCTGTCTCGGGCATAGGCCACTAGTGGATCTG  ATTGCAGTACCAGTCTTTCCC  GAGGCCCAGAATACCCTCCTTG  GGCTGGCAACTAATAGGGACACT |
| genR-1 | TCCAATTCAGCTGGCGTAATAGCGGCTATAATATCAGGTATACAGAATAT |
| genF-2 | CTGTATACCTGATATTATAGCCTGCTATTACGCCAGCTGAAT |
| genR-2 | GTGTACTAGAGGAGGCCAAGAGTAGCTGGAGCTCATAGCTTCA |
| genF-3 | TTTTGAAGCTATGAGCTCCAGCTACTCTTGGCCTCTCTAGT |
| genR-3 | CTGCGTACGGCCTGTTCGAAAGATCTACCACCGCTCTGGAAAGTGCCTCATCCAAAGGCGCAAATCCTG |
| genF-4 | CGCCCTATAGTGAGTCGTATTAC TGTTGGAATAAAAATCAACT |
| genR-4 | AAAAGGGAATCTGCAATTCT |
| genF-5 | TGTGAAATACCGCACAGATGCGT GAGCTCCAGCTGTTTATCA |
| genR-5 | GATAGTTGATTTTTATTCCAACA GTAATACGACTCACTATAG |
| genF-6 | CTACTGGCGCGTGGAGTAAAAAGGTTTGGATCAGGATTTGCGCCTTTGGA TGAGGCACTTTCCAGAGCG |
| genR-6 | ATAATGATAAACAGCTGGAGCTC ACGCATCTGTGCGGTATT |
| Miox4F | GCTTCTGAATGAGGTTGTTGACGAATC |
| Miox4R | AAGCCGTAAGATGCAGCCAATCC |
| UdhF | ATATACGAAGCTGCTAGAAGACATGGC |
| UdhR | ATGAATCTGGTCTACGTGGAGAATGC |
| PgkF | GTGCCAAGGTTGCTGACAAGATTC |
| PgkR | AAGCCATACCACCACCAATGATGATAG |

_
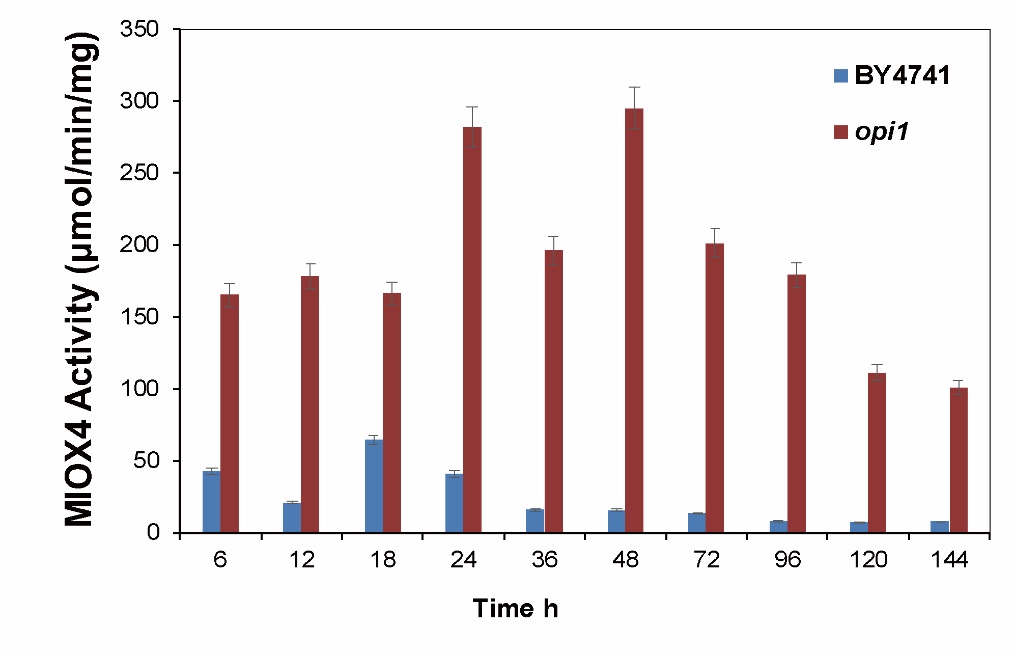
_

**Figure S1** MIOX4 activity of the episomal expression plasmid in the wild-type strain and *opi1* mutant strain with *myo*-inositol. All experiments were performed in triplicates and the error bar represented mean ± standard deviation.


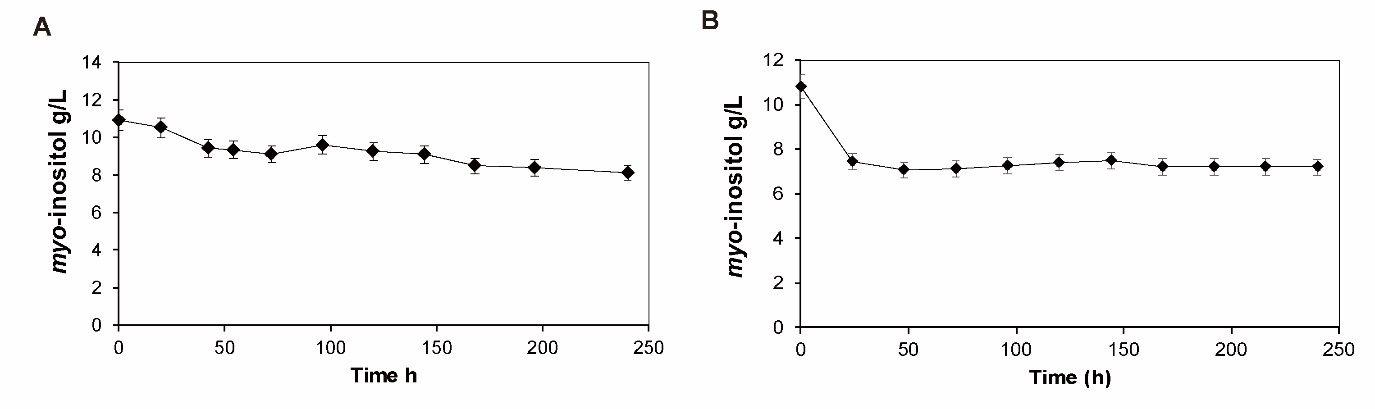


**Figure S2** *myo*-inositol residue in shake flask (**A**) and fed-batch (**B**) cultures when fed 60 mM (10.8 g/L) *myo*-inositol to the culture. All experiments were performed in triplicates and the error bar represented mean ± standard deviation.
